# Strain-Specific Liver Metabolite Profiles in Medaka

**DOI:** 10.3390/metabo11110744

**Published:** 2021-10-29

**Authors:** Hannah Soergel, Felix Loosli, Claudia Muhle-Goll

**Affiliations:** 1 Institute of Organic Chemistry, Karlsruhe Institute of Technology, Fritz-Haber-Weg 6, 76131 Karlsruhe, Germany; Hannah.Soergel@kit.edu; 2 Institute of Biological and Chemical Systems, Biological Information Processing (IBCS-BIP), Karlsruhe Institute of Technology, Hermann-von-Helmholtz-Platz 1, 76344 Eggenstein-Leopoldshafen, Germany; 3 Institute for Biological Interfaces 4 (IBG 4), Karlsruhe Institute of Technology, Hermann-von-Helmholtz-Platz 1, 76344 Eggenstein-Leopoldshafen, Germany

**Keywords:** liver, multivariate analysis, NMR spectroscopy, *Oryzias latipes*, univariate analysis

## Abstract

The relationship between genetic variation and phenotypic traits is often poorly understood since specific genotypes do not always easily translate into associated phenotypes, especially for complex disorders. The genetic background has been shown to affect metabolic pathways and thus contribute to variations in the metabolome. Here, we tested the suitability of NMR metabolomics for comparative analysis of fish lines as a first step towards phenotype-genotype association studies. The Japanese rice fish, medaka (*Oryzias latipes*), is a widely used genetic vertebrate model with several isogenic inbred laboratory strains. We used liver extracts of medaka iCab and HO5 strains as a paradigm to test the feasibility of distinguishing the metabolome of two different inbred strains. Fifteen metabolites could be detected in uni- and multivariate analyses that showed strain-specific levels. Differences could be assigned to specific metabolic pathways. Our results show that NMR spectroscopy is a suitable method to detect variance of the metabolome caused by subtle genetic differences. Thus, it has the potential to address genotype–phenotype associations in medaka, providing an additional level of phenotypic analysis.

## 1. Introduction

The advent of whole genome sequencing stimulated hope that the genetic variations in a population could be associated with pathology, especially for multi-causal diseases such as diabetes or cardiovascular diseases. The study of single gene mutations in the past forty years had revealed the impact of single genes and their protein products on disease etiology. Prominent examples include Huntington’s disease [1] or Duchenne muscular dystrophy [2]. In these cases, analysis of a particular gene by mutational or knockout studies led to a detailed understanding of the molecular mechanism underlying the fatal consequences of the respective disease. However, genetic studies have shown that many traits, including disease and its progression, are influenced by more than one gene, so called complex genetic traits [3]. Specifically, recent GWAS (genome wide association studies) revealed also a key influence of the genetic background on specific molecular traits such as the metabolome [4].

Metabolomic studies aim to identify and quantify comprehensively metabolites that are the products and/or effectors of cellular pathways. These studies are based on the assumption that the metabolic state of an organism provides crucial insight into its physiological state. For example, in many congenital metabolic disorders, the function of a single enzyme is perturbed and abnormal metabolite concentrations of the respective enzymatic reactions indicate a metabolomic disorder. Using this approach, characteristic metabolite patterns were detected in a multi-center clinical neonate metabolomics study in Turkey [5].

Furthermore, in cases where genomic approaches cannot reveal a unique genotype–phenotype correlation, differences in metabolites may correctly identify the pathological state. MODY5 is a specific form of maturity onset diabetes of the young, which causes multisystem disorder with a wide spectrum of clinical symptoms. Although more than 100 different mutations of the underlying causal gene, hepatocyte nuclear factor 1 homeobox B, have been reported, genomic approaches have not yet been able to reveal a genotype–phenotype correlation [6]. Metabolic differences in several tissues in a mouse model, however, clearly distinguished wild type from a mutant line [7]. That the metabolome highly depends on the genetic background is further demonstrated by studies of mouse strains. Distinct metabolite patterns of single organs such as the liver or brain [8], or physiological processes such as aging, [9] depended on the specific mouse strain employed in the study.

Teleost models such as medaka (*Oryzias latipes*) and zebrafish (*Danio rerio*) are widely used vertebrate models due to economical husbandry, high fecundity and short generation time. High evolutionary conservation of genes and their function permits using teleost models as disease models [10]. In contrast to mice, metabolomic studies of zebrafish are still sparse and only a few have been performed with medaka to date. Studies using zebrafish and medaka often aimed to analyze the impact of chemicals (e.g., [11,12]), environmental conditions [13] or daily or seasonal rhythms [14,15], and few are available that link changes in gene expression to changes of the metabolic profile [16].

Inbred strains are of particular importance as their fixed genomes permit experimental replication and analysis of how environmental factors impact on a given trait. Medaka offer a number of isogenic inbred strains, providing a paradigm to study the influence of the genomic background on the metabolism [10]. Despite the availability of several highly inbred medaka strains, previous studies did not include comparative studies using different strains to address this issue. Genotype–phenotype association studies of the metabolome require a sensitive methodology to reliably and quantitatively phenotype the metabolome of a high number of individuals. To test whether NMR spectroscopy fulfills these criteria, we analyzed whether strain specific metabolic differences can be detected in the medaka isogenic inbred strains iCab and HO5. Both strains are derived from the Southern Japanese population and their genomes therefore carry only minor differences [17]. Thus, for our test of the methodology, these strains provide an ideal setting to analyze whether strain specific differences based on genomic variation can be detected. As bioprobes, we used freshly dissected livers from adult fish.

We used NMR spectroscopy as a non-targeted analytical method, which allows the analysis of complex mixtures within a single experiment without prior elaborate processing. Although less sensitive than mass spectrometry, its excellent reproducibility, simple sample preparation and inherent quantitative nature make it perfectly suited for exploratory studies such as our comparative approach. Our analysis shows that strain-specific differences in the metabolic profile can be identified with high statistical power using liver extracts. Separation according to sex was less pronounced. The detection of explicitly distinct metabolic liver pattern by NMR spectroscopy shows that our analytical methodology can be applied for future genotype–phenotype studies of the metabolome in medaka.

## 2. Results

### 2.1. NMR Spectroscopy of Liver Extracts

Upon dissecting the livers, we observed that livers from the inbred strain HO5 were generally bigger than those of iCab. Similarly, livers of female fish were larger than their male counterparts within both strains, but since it was essential to immediately suppress metabolic pathways, livers could not be weighed after dissection, but were immediately snap frozen and lyophilized. Because of the variable sizes of livers, spectra showed different overall intensity ranges (Supplemental Table S1). However, our analysis of fish body length did not reveal a direct correlation of body length and liver size (Supplemental Table S1). Currently, no studies have been reported that analyzed the dependence of organ size on body size or body weight in medaka.

**Figure 1:**
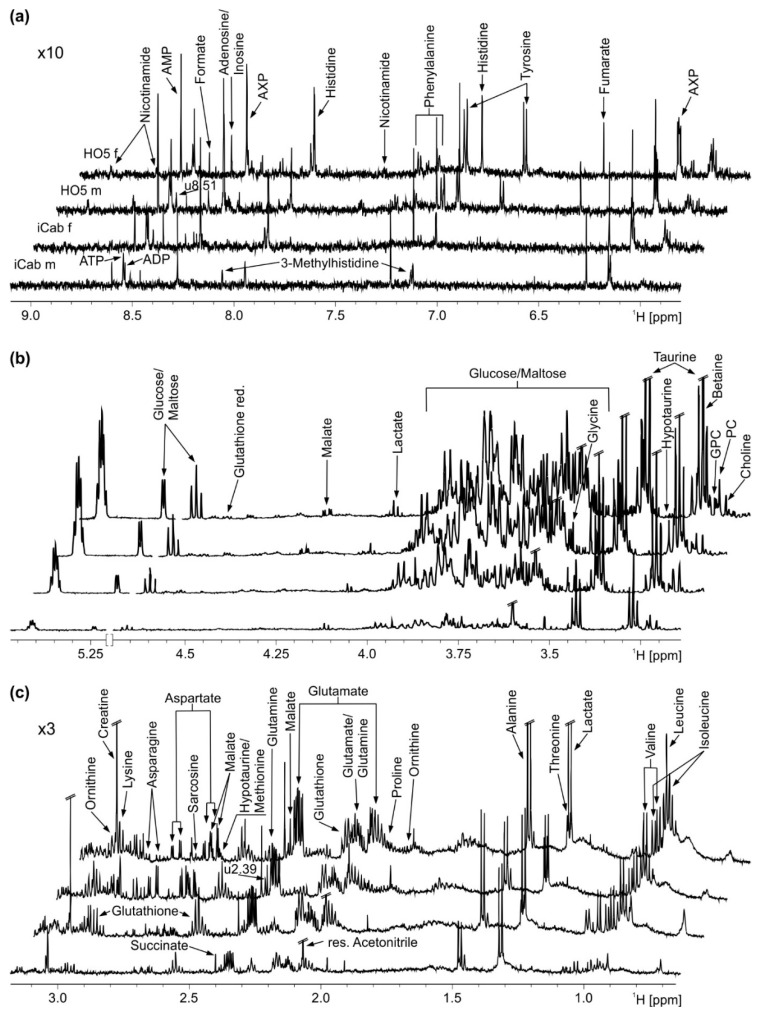
Representative ^1^H NMR spectra before normalization with annotations for all identified metabolites. (**a**) Aromatic region. (**b**) Sugar region. (**c**) Aliphatic region. From top to bottom: HO5 female (f), HO5 male (m), iCab female, iCab male. AXP: AMP, ADP and ATP, GPC: Glycerophosphocholine, PC: Phosphorylcholine.

Four representative spectra of male and female medaka fish of the inbred strains HO5 and iCab are shown in Figure 1. For statistical analysis, general peaks common to all individuals were selected using variable bucketing as integration mode. This excluded, e.g., resonances of bile acids such as cholic acid that were only present in a few samples. To take the observed liver size difference into account (that would otherwise confound the analysis), spectra were normalized to total intensity.

### 2.2. Strain-Specific Metabolic Profile of Liver Extracts

**Figure 2:**
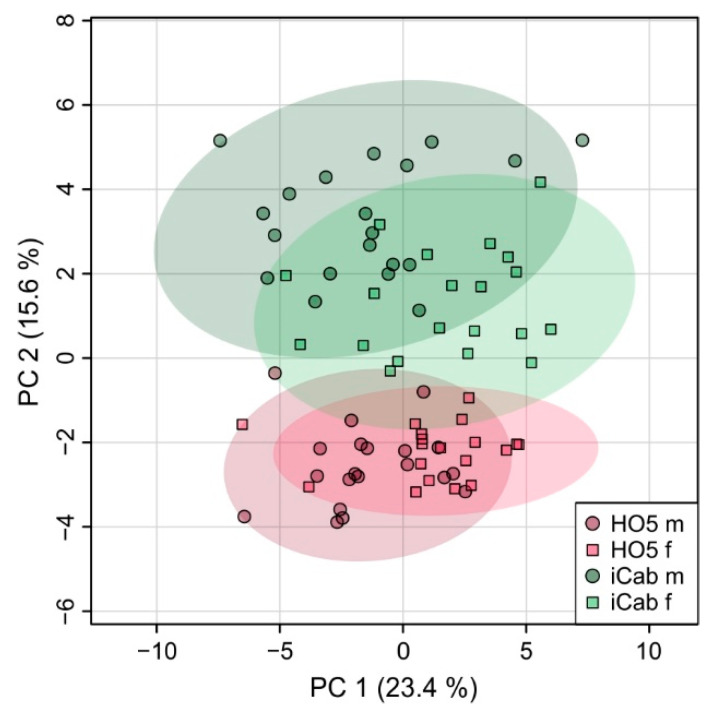
Principal component analysis comparing metabolic profiles of all samples, classified into HO5 male, HO5 female, iCab male and iCab female.

A clear separation between the strains was already observed in a principal component analysis (PCA) which does not use knowledge of group membership for clustering (Figure 2). Strains were separated predominantly along PC2, which explained 15.6% of the variation. Obviously, sex was a second criterion that led to clustering, this time along PC1, yet less pronounced. To understand whether strain separation was more obvious in one of the sexes, male and female samples were also analyzed separately for strain separation. This procedure increased the clustering of the two inbred strains, but in a comparable way for both sexes (Supplemental Figure S1). The observed differences along PC1 between individuals of genetically very similar (i.e., isogenic inbred) fish are most probably due to stochastic effects that occur during the relatively long lifetime of the fish prior to experimental validation. Living organisms, such as the extremely complex vertebrates, are not only the product of their genes, but encounter minor stochastic effects, both of genomic and environmental origin, that result in individual variation even in genetically identical organisms. As a result, for example, the size and weight of the fish were not entirely uniform but included some variation (Supplemental Table S1).

**Figure 3:**
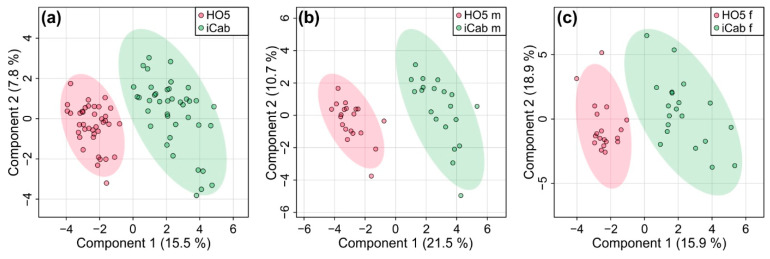
Partial least squares-discriminant analysis for discriminating the inbred strains HO5 and iCab (**a**) for male and female fish together and separated into (**b**) male and (**c**) female fish.

Partial least squares-discriminant analysis (PLS-DA) which, as a supervised method, takes group membership into account, revealed a clear separation between the two fish strains (Figure 3). Quality control parameters (R^2^: 0.88, Q^2^: 0.81) showed that the predictive power of the PLS-DA model was high. A 1000-fold permutation analysis resulted in a *p*-value < 0.001. Separation by sex did not alter this result, but females showed slightly lower quality parameters (R^2^: 0.88, Q^2^: 0.76, *p*-value < 0.001 for female, R^2^: 0.94, Q^2^: 0.86, *p*-value < 0.001 for male). Thus, the metabolic profile of liver could reliably distinguish the two inbred strains.

Metabolites responsible for the classification were identified using the variable importance in projection (VIP) scores of the PLS-DA analysis (Supplemental Figure S2, Table 1). The 15 most important metabolite buckets included aspartate, a bucket containing both lactate and threonine, adenosine triphosphate (ATP), an unknown substance with a resonance at 8.51 ppm, fumarate, phenylalanine, tyrosine and hypotaurine. In addition to these common features, some sex-specific differences between the inbred strains were observed.

We alternatively performed fold change (FC) analysis and two-sample *t*-tests as univariate analyses to identify differentially expressed metabolites between HO5 and iCab (FC > 1.3 or < 0.77 with an associated FDR (false discovery rate) adjusted *p*-value < 0.01). Analyzing all samples together resulted in smaller *p*-values than when male and female fish were separately analyzed (Table 1, a full list of all metabolites is given in Supplemental Table S2). One plausible reason for this result is that larger sample numbers in general achieve lower *p*-values. Moreover, analysis of female fish revealed *p*-values that were about an order of magnitude higher than for their male counterparts, and only 8 metabolite buckets showed significant *p*-values (*p* < 0.01), compared to 21 for male fish (data not shown). Therefore, a less stringent *p*-value criterion (*p* < 0.05) was used for female fish.

The most significant metabolites identified in this way overlapped with the most important metabolites identified in the PLS-DA models (Table 1). In particular, concentrations of the amino acids aspartate, ornithine, phenylalanine and tyrosine, and the dicarboxylic acid fumarate, as well as hypotaurine, were increased in HO5. Hypotaurine showed the highest fold changes (FC = 5–6), but when considering only female fish, the *p*-value was significantly higher than for their male counterparts (*p* = 0.04 compared to *p* < 0.0001). In contrast, only concentrations of lactate/threonine, ATP and an unknown substance with its resonance at 8.51 ppm, which is presumably also a nucleotide, were lower in HO5. In addition, some minor sex-specific differences in metabolite concentrations were found between the two inbred strains. Levels of malate and adenosine monophosphate (AMP) were elevated mainly in female HO5, whereas sarcosine and choline levels were lower. In contrast, alanine levels were decreased only in male HO5.

### 2.3. Metabolic Differences between Male and Female Fish Are Not as Pronounced as between the Inbred Strains

**Figure 4:**
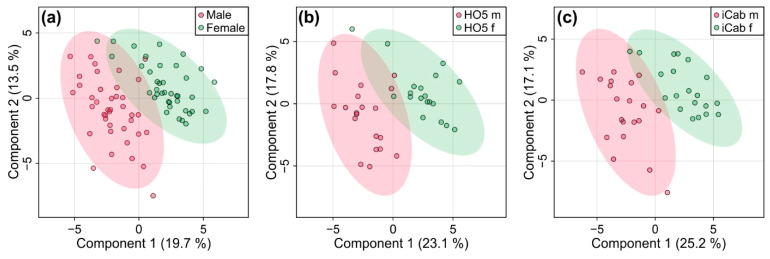
Partial least squares-discriminant analysis for discriminating male and female fish (**a**) for HO5 and iCab together and separated into (**b**) HO5 and (**c**) iCab.

Since PCA analysis had revealed additional clustering according to sex, we also looked at sex-specific differences in the metabolome of the fish for comparison by PLS-DA (Figure 4). Quality control parameters (R^2^: 0.93 and Q^2^: 0.67, *p* < 0.001) showed that the model was reliable. When sex impact was analyzed separately for HO5 and iCab, separation could be further enhanced by PLS-DA. Quality control parameters showed that the metabolic profile could reliably distinguish male and female fish of the same strain. (R^2^: 0.87 and Q^2^: 0.58 for HO5, R^2^: 0.91 and Q^2^: 0.75 for iCab). A 1000-fold permutation analysis resulted in *p*-values of 0.002 and 0.001, respectively. However, the separation was not as pronounced as the one we observed between strains (Figure 3).

Among the 15 most important metabolites in each PLS-DA model (Supplemental Figure S3, Table 2), only 4 occurred in both strains, namely: 3-methylhistidine, creatine (both VIP-scores > 1.8), reduced glutathione and tyrosine. In addition, some strain-specific differences between male and female fish could be observed.

Using univariate analysis, we compared the metabolite levels between male and female fish. Here, most of the metabolites with fold changes >1.3 or <0.77 with an associated FDR adjusted *p*-value < 0.01 corresponded to the most important metabolites according to each PLS-DA model (Table 2, a full list of all metabolites is given in Supplemental Table S3). Significantly elevated metabolites in males were 3-methylhistidine, with a fold change between 12 and 13, and an unknown substance with its resonance at 8.51 ppm. Less abundant metabolites were creatine and tyrosine. When a less stringent *p*-value criterion (*p* < 0.05) was used, levels of nicotinamide also appeared to be higher, and levels of reduced glutathione, ornithine, hypotaurine and formate lower in male fish. Moreover, strain-specific differences between male and female were observed. For example, levels of glutamine and alanine were decreased only in males of the HO5 strain. When considering only iCab, glycerophosphocholine and ATP were increased and aspartate decreased in males.

## 3. Discussion

As one of the main “omics-” approaches, metabolomics—the comprehensive study of metabolites within cells, tissues, or body fluids—is important for the analysis of physiological processes. Metabolic pathways are complex interplays involving the activity of many genes. Changes in cellular activities are directly translated into altered metabolite patterns. Importantly, in addition to the genes encoding metabolic enzymes, the genetic background also directly influences the metabolome, especially when the metabolite pattern of single organs such as livers or brains were investigated, as was demonstrated in metabolome studies of mouse strains [8]. Similar comparative inter-strain studies have not been reported for teleost laboratory models. Thus, our aim was to examine whether an NMR metabolomics approach could serve as a methodology to answer the question of how the genetic background influences the metabolome in medaka isogenic inbred strains.

It has previously been shown that sex-specific differences of the medaka metabolome can be detected [18]. The aim of our work was to test whether NMR spectroscopy as an analytical tool can discriminate the metabolome of different medaka inbred strains. This will, on the one hand, permit the study of specific genes in metabolic pathways using mutational approaches, but importantly, it also provides the means for future genotype–phenotype associations. To establish a proof of principle, we focused on the liver metabolome of the isogenic inbred medaka strains iCab and HO5. We selected these strains as they are both derived from the Southern population and therefore carry only subtle genetic variations [17]. These strains thus provide an ideal setting to test whether NMR spectroscopy can detect strain specific differences of the metabolome. In vertebrates, the liver is the main metabolic organ, and hepatic metabolites and transcripts reflect the organismal physiological state [19]. We applied a biphasic acetonitrile/water/chloroform extraction protocol to quantify small polar metabolites from liver extracts of the strains iCab and HO5. We used NMR spectroscopy as the analytical method because the easy sample preparation, combined with excellent reproducibility, render NMR unrivaled for explorative purposes.

Both multivariate and univariate statistical analysis showed that strain-specific metabolic states could be identified with high statistical power in medaka liver extracts. We determined a panel of 15 metabolites that distinguish the 2 strains.

**Figure 5:**
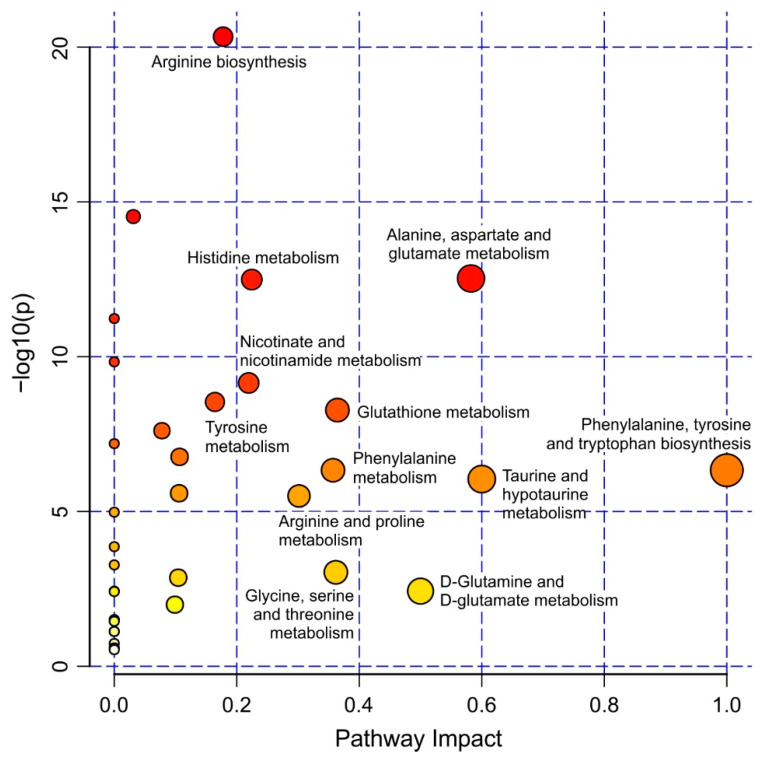
Pathway analysis revealing altered metabolic pathways between HO5 and iCab based on pathway impact score and *p*-value. Pathways having pathway impact > 0.2 and −log(*p*) > 2 were labelled. Pathway analysis was performed with the metabolites listed in Table S2.

Interestingly, several of the metabolites with strain-specific relative amounts are key molecules of specific metabolic pathways in the liver [20]. Pathway analysis performed within MetaboAnalyst (Figure 5), also revealed several affected ones. Aspartate, fumarate, and ornithine are metabolites of the urea cycle which converts toxic ammonia into urea in the liver. In KEGG the urea cycle is part of the arginine biosynthesis and one specific step of the urea cycle, the conversion of aspartate to fumarate is also part of the alanine, aspartate and glutamate metabolism. Our analysis shows that aspartate, fumarate, and ornithine were all present at elevated relative amounts in the HO5 strain, suggesting a misregulation of this important pathway in HO5.

Elevated levels of aspartate and malate in HO5 could also indicate an affected malate aspartate shuttle in the mitochondria. ATP and AMP levels are connected to both pathways, but as liver preparation was time-consuming and we did not take explicit precautions to quench enzymatic reactions, these two metabolites are probably less significant. Fumarate is also involved in the phenylalanine degradation pathway. Interestingly, both phenylalanine and tyrosine levels were higher in HO5 fish. Tyrosine, on the other hand, is an intermediate metabolite of melanin synthesis. HO5 fish show reduced melanin pigmentation (F. Loosli, unpublished observation). We therefore speculate that melanin synthesis is affected in this inbred strain, leading to an accumulation of the upstream intermediates of melanin synthesis. Sarcosine and choline, which are both lower in HO5, could indicate a distinct hepatic methylamine osmolyte metabolism in this strain. Striking is the enormous fold change of hypotaurine observed for both sexes in HO5. Hypotaurine is an intermediate in the biosynthesis of taurine. Lactate, on the other hand, is severely reduced in HO5, which hints at an altered gluconeogenesis/cori cycle in liver. Thus, our comparative metabolome analysis identifies strain-specific changes of key metabolites between strains. Future experiments will be designed to test our hypothesis concerning affected metabolic pathways.

As PCA revealed that male and female samples showed some variation, we also analyzed the sexes separately for strain-separating metabolites. This allowed us to assess the robustness of the method. Overall, however, the same metabolites contributed to the statistical separation of the strains, regardless of whether sex was taken into account or not. Very few extra metabolites stood out that were different in iCab or HO5 when male and female group membership was used as additional criterion.

To prove that the identified metabolite pattern was truly strain-specific, we assessed whether the liver extracts could also be grouped according to sex only. Among the 14 metabolites that contributed to sex differentiation, 50% were entirely different from the ones that separated the strains. The most striking one was 3-Methylhistidine, with a fold change between 12 and 13 in male liver, followed by creatine. Among the metabolites that already showed up for strain separation, hypotaurine levels were elevated in females. Taken together, separation on the basis of sex was possible, but yielded an inferior separation with lower quality values than a separation on the basis of strain. This was surprising given that the requirements for egg production in females should lead to marked different metabolic requirements in the liver. Our findings are in good agreement with published data, reporting sex-specific differences in metabolite patterns in rodents [21,22,23], humans [24] and also medaka, the latter using a multi-omics approach [18].

Genetic screening, performed with the aim to understand the association of genotype and phenotype, has experienced a dramatic increase in the past years due to ever lower genome sequencing costs. However, often when comparing specific genotypes the associated phenotypes differ in subtle ways. Expression analyses at the mRNA level also give only an approximation of gene function, as they do not, per se, allow us to assess the effect on the organism as mRNA stability and/or protein translation product or further regulations also determine the activity of a gene product. Thus, metabolomics—the comprehensive study of small molecules within cells, tissues, or body fluids—provides important complementary insights. In summary, we show that NMR spectroscopy of adult liver tissue can detect quantitative strain specific differences in medaka fish. Therefore, our approach provides a tool to address genotype–phenotype associations in this vertebrate model system that will further our understanding of how the metabolome is influenced by the genetic background.

## 4. Materials and Methods

### 4.1. Animal Handling and Tissue Collection

The inbred, isogenic medaka strains iCab and HO5 were kept in closed stocks at the Institute of Biological and Chemical Systems, Biological Information Processing (IBCS-BIP) at the Karlsruhe Institute of Technology (KIT), as previously described in recirculatory systems, under 14 h light/10 h dark conditions at 26 °C [25]. Fish husbandry was performed in accordance with EU directive 2010/63/EU guidelines as well as with German animal protection regulations (Tierschutzgesetz §11, Abs. 1, no. 1; Regierungspräsidium Karlsruhe, Germany; husbandry permits AZ35-9185.64/BH KIT). The fish facilities are under the supervision of the Regierungspräsidium Karlsruhe, who approved the experimental procedures.

All fish used for this study were fed the same way: equal amounts of dry flakes (Tetramin flakes, TETRA) were fed in the morning and afternoon, equal amounts of fresh *Artemia* nauplii were fed at noon. Seven month old fish of both sexes were used for this study. Fish were divided into four groups according to inbred strain and sex. A group size of 20 fish was chosen (19 for HO5 female), resulting in a total of 79 fish. After fish were sacrificed by hypothermic shock, individual livers were immediately dissected and collected in empty homogenization tubes (Precellys Lysing KITS CK 14, Berting GmbH, Frankfurt (Main), Germany). Samples were placed on dry ice for transport, subsequently lyophilized and stored at −25 °C until extraction.

### 4.2. Sample Preparation

Based on an established extraction protocol [16], we developed a biphasic acetonitrile/water/chloroform extraction method (ratio 1:1:1) for separating polar from non-polar metabolites. Amounts of 1 mL of cold acetonitrile/water (1:1), 300 µL of cold chloroform and 1.4 mm ceramic beads were added to each sample and vortexed for 1 min. Tissue was homogenized using a liquid nitrogen cooled homogenizer (Precellys Evolution Homogenizer with Cryolys Cooling Option, Berting GmbH, Frankfurt (Main), Germany). Homogenization was performed at 6000 rpm and 4 °C. A total of four cycles were completed, each lasting 20 s followed by a waiting time of 120 s. The homogenates were incubated at 4 °C for 10 min and then transferred to new micro centrifuge tubes. Next, remaining chloroform (200 µL) was added, samples were vortexed for 1 min and left at 4 °C for 10 min to partition. Samples were then centrifuged for 10 min at 21,382 g and 4 °C. Finally, upper polar layers were carefully removed into new micro centrifuge tubes and lyophilized overnight. All samples were kept at −25 °C until performing NMR experiments.

Quality control (QC) samples for ^1^H-NMR spectroscopy were prepared containing six metabolites commonly found in tissues. The quality control contained 1 mM glucose and 0.5 mM each of the following: valine, glutamate, choline, acetate and phenylalanine, which were dissolved in the same phosphate buffer as the tissue samples. Aliquots of QC samples were kept at −25 °C until measurement.

### 4.3. ^1^H-NMR Spectroscopy of Liver Samples

Aqueous phase extracts were dissolved in 700 µL phosphate buffer (0.15 M in D_2_O containing 0.6 mM trimethylsilylpropanoic acid (TSP) as an internal standard and 0.2 mM sodium azide, pH 7.2). The samples were centrifuged for 10 min at 21,382 g and 4 °C. A total of 600 µL were then transferred to a 5-mm NMR tube and analyzed immediately by ^1^H-NMR. ^1^H NMR spectra were acquired using a Bruker Avance II + 600 MHz spectrometer (Bruker Biospin, Rheinstetten, Germany) equipped with a double resonance 5-mm BBI probe or a triple resonance broad band 5-mm TBI probe. Temperature was calibrated to 300 K on a daily basis. QC samples were included once a day. One-dimensional spectra were recorded using the Bruker pulse sequence noesygppr1D with 64 k data points, 512 transients and a relaxation delay of 4 s over a spectral width of 20 ppm. Water suppression was achieved through presaturation with a selective pulse B1 field strength of 25 Hz. Prior to Fourier transformation, spectra were multiplied with an exponential function with a 0.3 Hz line broadening factor. NMR spectra were automatically phased and baseline corrected and manually referenced to internal TSP (δ 0.0) using Topspin (Bruker Biospin, Rheinstetten, Germany). In addition, 2D ^1^H-^1^H correlation spectroscopy (COSY), and ^1^H-^13^C heteronuclear single-quantum correlation (HSQC) experiments were recorded for identification and assignment of metabolites.

### 4.4. Data Analysis

NMR spectra were analyzed for bucketing and annotation using MestReNova 14.2 (Mestrelab Research S.L., Santiago de Compostela, Spain). Metabolites were assigned based on the Human Metabolome Database (HMDB) and the evaluation version of Chenomx NMR Suite 8.5 (Chenomx, Edmonton, Canada). To confirm the assignments, representative samples were spiked with small amounts of the particular substances. Spectra were divided into variable sized buckets leading to 46 buckets that contained only signals of one specific metabolite whenever possible. Some of the metabolites were characterized by separate buckets for protons of different functional groups. Glucose was not included in the analysis as we noticed some increase in its intensity when we repeated measurements, which was probably due to residual glycogen degradation. A table with all ppm values can be found in the Supporting Information (Table S2).

Statistical analysis was performed using MetaboAnalyst 5.0 [26]. Prior to data analysis, spectra of blank buffer were subtracted. Data were normalized to total intensity and auto scaled to take into account that the amount of tissue varied between individual samples, generating a Gaussian distribution plot.

To observe intrinsic metabolic variations, multivariate analyses including principal component analysis (PCA) and partial least squares-discriminant analysis (PLS-DA) were performed. To assess the quality of the resulting PLS-DA models, permutation testing with 1000 permutations, as well a 10-fold internal cross-validation, were performed. The predictive ability of the models was judged by the quality parameters Q^2^ (quality of prediction) and R^2^ (explained variance). Between-group comparisons were performed using univariate fold change (FC) analysis. Student’s *t*-tests were performed to determine statistical significance in MetaboAnalyst which yield FDR adjusted *p*-values based on the Benjamini–Hochberg procedure. We used the KEGG database for pathway analysis in metaboanalyst [27].

## Figures and Tables

**Table 1:** Top 15 metabolites contributing to the variance between HO5 and iCab according to PLS-DA and FC analysis. Analyses were performed separately for male and female fish, as well as with both sexes together. Only metabolite buckets are shown that had VIP values > 1.2 and FC > 1.3 or <0.77 for at least one of the different groups. For each FC, the associated FDR-adjusted *p*-value is shown.

	Both Sexes		Male		Female	
	Multivariate	Univariate	Multivariate	Univariate	Multivariate	Univariate
	VIP Score	FC (*p*-Value)	VIP Score	FC (*p*-Value)	VIP Score	FC (*p*-Value)
Aspartate	1.86	1.54 (1.39 × 10^−9^)	1.79	1.70 (1.62 × 10^−7^)	1.67	1.41 (0.0012)
Lactate/Threonine	1.82	0.56 (2.88 × 10^−9^)	1.57	0.46 (1.51 × 10^−5^)	1.87	0.67 (1.16 × 10^−4^)
ATP	1.80	0.56 (2.90 × 10^−9^)	1.71	0.48 (9.37 × 10^−7^)	1.65	0.67 (0.0012)
u8.51	1.66	0.54 (1.08 × 10^−7^)	1.48	0.53 (5.01 × 10^−5^)	1.91	0.53 (1.16 × 10^−4^)
Fumarate	1.58	2.21 (6.94 × 10^−7^)	1.52	3.09 (2.77 × 10^−5^)	1.29	1.69 (0.0170)
Phenylalanine	1.35	1.65 (5.40 × 10^−5^)	1.30	2.00 (6.78 × 10^−4^)	1.09	1.39 (0.0428)
Malate	1.29	1.34 (1.42 × 10^−4^)	0.96	1.32 (0.0154)	1.44	1.37 (0.0066)
Ornithine	1.22	1.34 (2.78 × 10^−4^)	1.06	1.35 (0.0073)	1.46	1.34 (0.0066)
Tyrosine	1.22	1.49 (2.78 × 10^−4^)	1.23	1.61 (0.0013)	1.35	1.43 (0.0118)
Hypotaurine	1.20	5.10 (3.54 × 10^−4^)	1.57	5.69 (1.51 × 10^−5^)	1.10	5.09 (0.0428)
Alanine	1.19	0.78 (3.70 × 10^−4^)	1.52	0.72 (2.77 × 10^−5^)	0.90	0.83 (0.0971)
u2.39	1.15	1.40 (5.39 × 10^−4^)	1.30	1.88 (6.78 × 10^−4^)	0.56	1.10 (0.3076)
AMP	1.14	1.36 (5.40 × 10^−4^)	0.85	1.39 (0.0318)	1.43	1.33 (0.0066)
Choline	1.06	0.79 (0.0013)	0.72	0.87 (0.0741)	1.28	0.72 (0.0170)
Sarcosine	1.02	0.77 (0.0022)	0.45	0.88 (0.3004)	1.42	0.69 (0.0066)

**Table 2:** Top 14 metabolites contributing to the variance between male and female fish according to PLS-DA and FC analysis. Analyses were performed separately for HO5 and iCab fish, as well as with both strains together. Only metabolite buckets are shown that had VIP values > 1.2 and FC > 1.3 or <0.77 for at least one of the different groups. For each FC, the associated FDR-adjusted *p*-value is shown.

	Both Strains		HO5		iCab	
	Multivariate	Univariate	Multivariate	Univariate	Multivariate	Univariate
	VIP Score	FC (*p*-Value)	VIP Score	FC (*p*-Value)	VIP Score	FC (*p*-Value)
3-Methylhistidine	2.31	12.77 (2.63 × 10^−12^)	2.10	12.48 (1.31 × 10^−6^)	1.84	13.00 (1.46 × 10^−5^)
Creatine	2.28	0.42 (3.98 × 10^−12^)	1.84	0.42 (9.39 × 10^−5^)	2.06	0.42 (1.64 × 10^−7^)
Glutathione_red	1.68	0.65 (1.11 × 10^−5^)	1.33	0.76 (0.0114)	1.55	0.55 (0.0006)
Glutamine	1.57	0.80 (5.15 × 10^−5^)	1.83	0.75 (9.39 × 10^−5^)	0.94	0.85 (0.0579)
Tyrosine	1.47	0.66 (0.0002)	1.49	0.69 (0.0037)	1.32	0.61 (0.0053)
Glycerophosphocholine	1.45	1.39 (0.0002)	0.82	1.19 (0.1397)	1.57	1.60 (0.0005)
Ornithine	1.36	0.75 (0.0006)	1.23	0.75 (0.0145)	1.61	0.75 (0.0003)
Hypotaurine	1.31	0.20 (0.0010)	1.05	0.20 (0.0433)	1.72	0.18 (8.43 × 10^−5^)
Formate	1.29	0.16 (0.0010)	1.07	0.20 (0.0394)	1.27	0.16 (0.0061)
Nicotinamide	1.28	1.57 (0.0010)	1.27	1.56 (0.0125)	1.04	1.59 (0.0343)
Alanine	1.19	0.80 (0.0022)	1.51	0.74 (0.0033)	0.79	0.86 (0.1132)
u8.51	1.16	1.46 (0.0028)	1.36	1.47 (0.0091)	1.27	1.47 (0.0061)
Aspartate	0.75	0.86 (0.0743)	0.52	0.92 (0.4048)	1.30	0.76 (0.0058)
ATP	0.74	1.23 (0.0749)	0.03	1.01 (0.9656)	1.33	1.39 (0.0053)

## Data Availability

The datasets used and analyzed during the current study are available from the corresponding author on reasonable request.

## Supplementary Materials

The following are available online at https://www.mdpi.com/article/10.3390/metabo11110744/s1, Figure S1: Principal component analysis (PCA) of the two inbred strains HO5 and iCab, Figure S2: Variable importance in projection (VIP) plot presenting the relative contribution of the 15 most important metabolite features to the variance between HO5 and iCab, Figure S3: Variable importance in projection (VIP) plot presenting the relative contribution of the 15 most important metabolite features to the variance between male and female fish, and Table S1: Fish body length and body weight. The original weight of the livers is reflected by the intensity of the spectra, Table S2: Complete list of buckets with their FC values and *p*-values for comparison between HO5 and iCab. For all buckets, the respective chemical shift range is shown, Table S3: Complete list of buckets with their FC values and *p*-values for comparison between male and female fish. For all buckets, the respective chemical shift range is shown.
