# Supplementary material for: Strain-Specific Liver Metabolite Profiles in Medaka
Source: Metabolites. 2021 Oct 29;11(11):744. doi: 10.3390/metabo11110744 (PMC8617739; doi:10.3390/metabo11110744)
Supplement: Supplementary file 1 [file metabolites-11-00744-s001.zip › metabolites-1400974-supplementary.pdf]

Strain-specific liver metabolite profiles in Medaka

Hannah Soergel [1], Felix Loosli [2,*] and Claudia Muhle-Goll [3,*]

[1] Institute of Organic Chemistry, Karlsruhe Institute of Technology, Fritz-Haber-Weg 6, 76131 Karlsruhe, Germany; Hannah.Soergel@kit.edu
[2] Institute of Biological and Chemical Systems, Biological Information Processing (IBCS-BIP), Karlsruhe Institute of Technology, Hermann-von-Helmholtz-Platz 1, 76344 Eggenstein-Leopoldshafen, Germany
[3] Institute for Biological Interfaces 4 (IBG 4), Karlsruhe Institute of Technology, Hermann-von-Helmholtz-Platz 1, 76344 Eggenstein-Leopoldshafen, Germany
* Correspondence: Felix.Loosli@kit.edu (F.L.); Claudia.Muhle-Goll@kit.edu (C.M.-G.); Tel.: +49-72160828743 (F.L.); +49-72160829357(C.M.-G.)

Supplemental Information

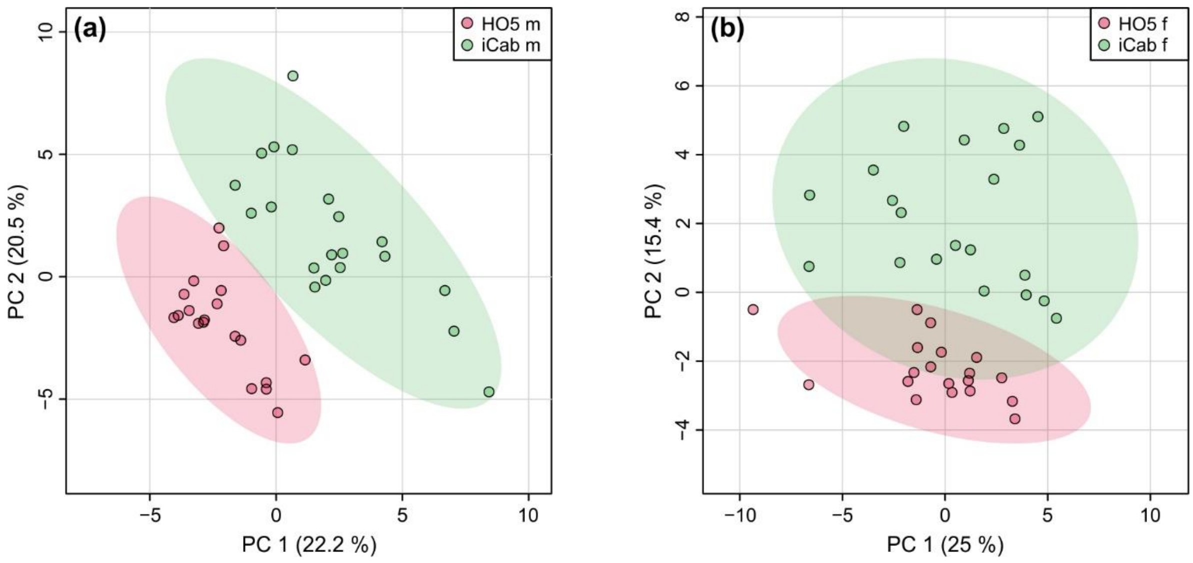

Figure S1. Principal component analysis (PCA) of the two inbred strains HO5 and iCab. (**a**) Male fish. (**b**) Female fish.

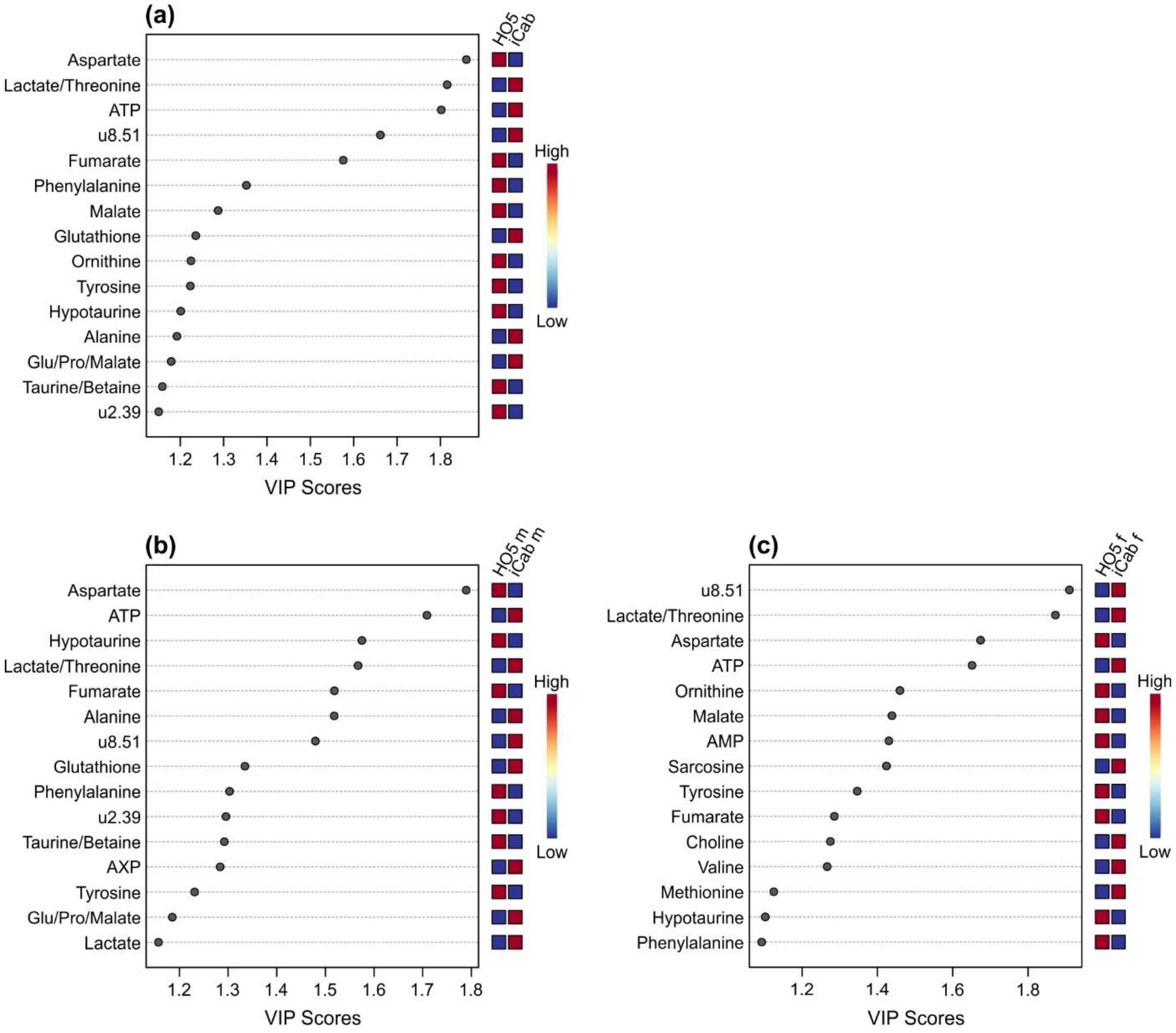

Figure S2. Variable importance in projection (VIP) plot presenting the relative contribution of the 15 most important metabolite features to the variance between HO5 and iCab. (**a**) Male and female fish together. (**b**) Male fish. (**c**) Female fish.

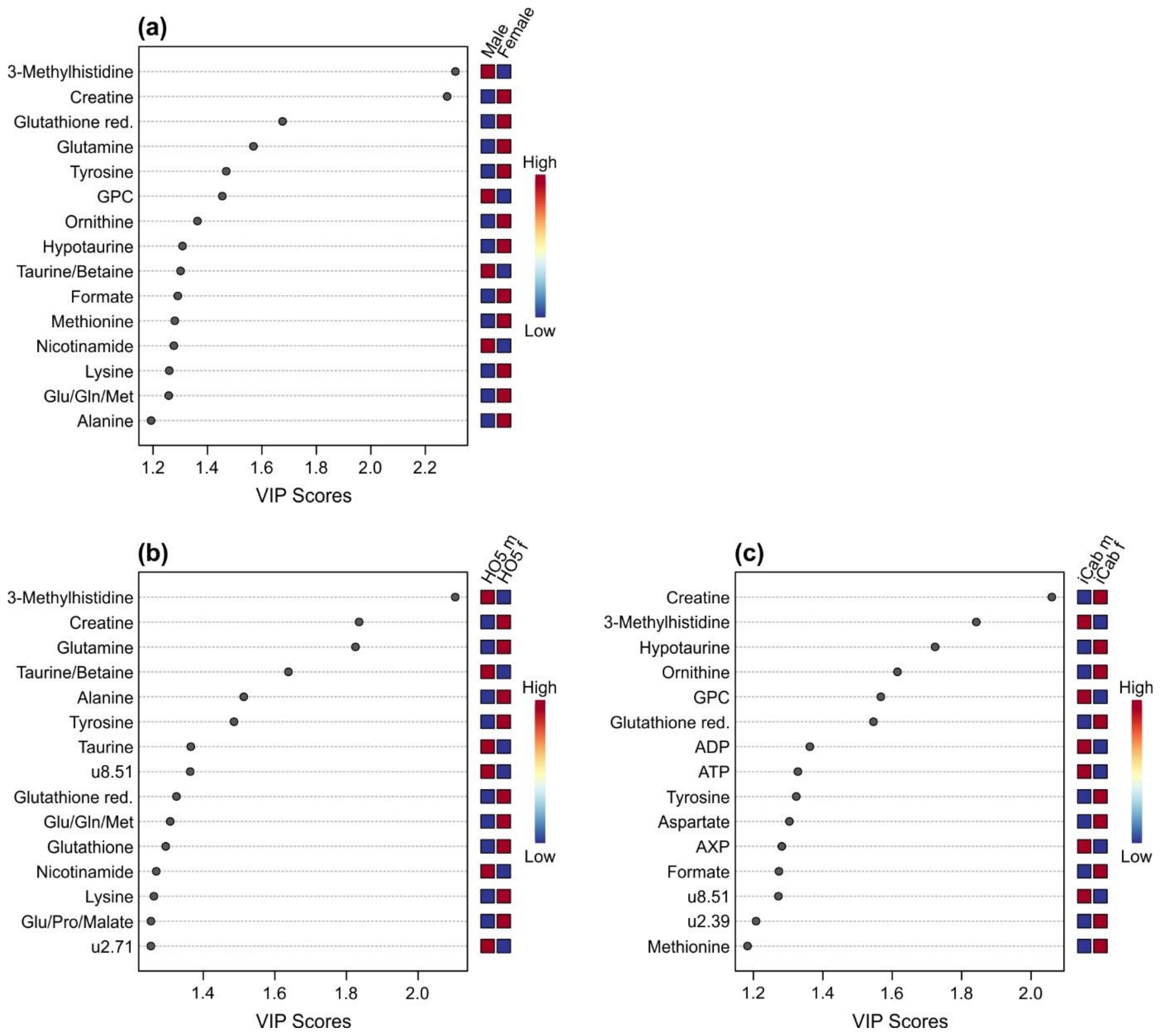

Figure S3. Variable importance in projection (VIP) plot presenting the relative contribution of the 15 most important metabolite features to the variance between male and female fish. (**a**) HO5 and iCab together. (**b**) HO5. (**c**) iCab.

Table S1. Fish body length and body weight. The original weight of the livers is reflected by the intensity of the spectra. Spectrum intensities were normalized to the TSP signal.

	Body Length [cm]		Body Weight [mg]		Spectrum Intensity	
Group	Mean	Standard Deviation	Mean	Standard Deviation	Mean	Standard Deviation
**HO5 Female**	2.77	0.19	280	49	4.23	1.93
**HO5 Male**	2.83	0.13	288	52	3.66	2.17
**iCab Female**	2.86	0.28	306	82	2.56	1.19
**iCab Male**	3.27	0.23	445	89	1.04	0.48

Table S2. Complete list of buckets with their FC values and *p*-values for comparison between HO5 and iCab. For all buckets, the respective chemical shift range is shown. Abbreviations: Glu: Glutamate, Pro: Proline, Gln: Glutamine, Met: Methionine, ATP: Adenosine triphosphate, ADP: Adenosine diphosphate, AMP: Adenosine monophosphate, AXP: AMP, ADP and ATP.

		Both Sexes		Male		Female	
**Bucket**	**Chemical Shift Range [ppm]**	**FC**	***p*-Value (FDR corr.)**	**FC**	***p*-Value (FDR corr.)**	**FC**	***p*-Value (FDR corr.)**
Leucine	0.96 – 0.98	1.08	2.22×10^{-1}	1.10	3.53×10^{-1}	1.05	5.26×10^{-1}
Isoleucine	1.01 – 1.03	0.92	1.79×10^{-1}	0.98	8.50×10^{-1}	0.87	7.64×10^{-2}
Valine	1.03 – 1.06	0.87	2.14×10^{-2}	0.92	4.16×10^{-1}	0.82	1.70×10^{-2}
Lactate/Threonine	1.32 – 1.35	0.56	2.88×10^{-9}	0.46	1.51×10^{-5}	0.67	1.16×10^{-4}
Alanine	1.47 – 1.50	0.78	3.70×10^{-4}	0.72	2.77×10^{-5}	0.83	9.71×10^{-2}
Proline	1.99 – 2.02	0.96	4.93×10^{-1}	0.97	8.33×10^{-1}	0.95	4.87×10^{-1}
Glu/Pro	2.02 – 2.07	0.89	1.01×10^{-2}	0.85	5.70×10^{-2}	0.92	1.14×10^{-1}
Glu/Gln/Met	2.10 – 2.14	0.87	1.18×10^{-3}	0.86	9.97×10^{-3}	0.89	4.28×10^{-2}
Glu/Pro/Malate	2.33 – 2.38	0.83	4.16×10^{-4}	0.78	2.19×10^{-3}	0.89	8.44×10^{-2}
u2.39	2.39 – 2.40	1.40	5.39×10^{-4}	1.88	6.78×10^{-4}	1.10	3.08×10^{-1}
Succinate	2.40 – 2.41	0.91	1.60×10^{-1}	0.82	5.70×10^{-2}	1.02	7.96×10^{-1}
Glutamine	2.44 – 2.48	0.93	1.79×10^{-1}	0.87	1.54×10^{-2}	0.99	8.77×10^{-1}
Glutathione	2.51 – 2.60	0.85	2.78×10^{-4}	0.83	5.01×10^{-4}	0.88	6.71×10^{-2}
Methionine	2.64 – 2.65	0.83	1.35×10^{-3}	0.84	9.97×10^{-3}	0.83	4.28×10^{-2}
Malate	2.65 – 2.66	1.34	1.42×10^{-4}	1.32	1.54×10^{-2}	1.37	6.61×10^{-3}
u2.71	2.71 – 2.72	1.15	3.82×10^{-2}	1.32	4.92×10^{-3}	0.98	7.96×10^{-1}
Sarcosine	2.74 – 2.74	0.77	2.15×10^{-3}	0.88	3.00×10^{-1}	0.69	6.61×10^{-3}
u2.76	2.75 – 2.76	1.19	6.49×10^{-3}	1.24	1.54×10^{-2}	1.15	1.52×10^{-1}
Aspartate	2.79 – 2.84	1.54	1.39×10^{-9}	1.70	1.62×10^{-7}	1.41	1.20×10^{-3}
Asparagine	2.85 – 2.89	1.09	8.16×10^{-2}	1.17	3.18×10^{-2}	1.02	7.96×10^{-1}
Lysine	3.01 – 3.03	1.08	1.60×10^{-1}	1.07	3.96×10^{-1}	1.10	2.42×10^{-1}
Creatine	3.03 – 3.04	0.97	9.04×10^{-1}	0.99	9.67×10^{-1}	0.98	8.77×10^{-1}
Ornithine	3.05 – 3.08	1.34	2.78×10^{-4}	1.35	7.27×10^{-3}	1.34	6.61×10^{-3}
Choline	3.20 – 3.21	0.79	1.32×10^{-3}	0.87	7.41×10^{-2}	0.72	1.70×10^{-2}
Phosphorylcholine	3.22 – 3.23	0.99	9.67×10^{-1}	0.83	3.39×10^{-1}	1.18	1.95×10^{-1}
Glycerophosphocholine	3.23 – 3.24	0.87	1.35×10^{-1}	0.77	3.66×10^{-2}	1.03	7.05×10^{-1}
Taurine/Betaine	3.27 – 3.28	1.13	5.20×10^{-4}	1.18	6.78×10^{-4}	1.08	1.20×10^{-1}
Hypotaurine	3.36 – 3.36	5.10	3.54×10^{-4}	5.69	1.51×10^{-5}	5.09	4.28×10^{-2}
Taurine	3.42 – 3.43	1.11	8.19×10^{-3}	1.14	8.80×10^{-3}	1.07	2.73×10^{-1}
Glycine	3.56 – 3.56	1.07	3.36×10^{-1}	1.04	7.05×10^{-1}	1.10	4.59×10^{-1}
Lactate	4.09 – 4.11	0.88	5.39×10^{-4}	0.84	2.89×10^{-3}	0.92	1.28×10^{-1}
u4.12/Lactate	4.11 – 4.12	0.93	1.35×10^{-1}	0.92	3.01×10^{-1}	0.93	2.73×10^{-1}
Glutathione_red	4.56 – 4.59	0.98	9.67×10^{-1}	1.19	2.45×10^{-1}	0.87	1.92×10^{-1}
Fumarate	6.52 – 6.52	2.21	6.94×10^{-7}	3.09	2.77×10^{-5}	1.69	1.70×10^{-2}
Histidine	7.11 – 7.12	0.94	2.07×10^{-1}	1.02	8.85×10^{-1}	0.86	4.28×10^{-2}
Tyrosine	7.18 – 7.22	1.49	2.78×10^{-4}	1.61	1.32×10^{-3}	1.43	1.18×10^{-2}
Phenylalanine	7.42 – 7.45	1.65	5.40×10^{-5}	2.00	6.78×10^{-4}	1.39	4.28×10^{-2}
3-Methylhistidine	8.05 – 8.06	1.00	9.67×10^{-1}	0.97	9.67×10^{-1}	1.01	9.50×10^{-1}
AXP	8.27 – 8.29	0.86	5.39×10^{-4}	0.80	7.02×10^{-4}	0.93	1.95×10^{-1}
Adenosine/Inosine	8.35 – 8.36	1.32	4.36×10^{-2}	1.59	3.18×10^{-2}	1.07	7.63×10^{-1}
Formate	8.46 – 8.46	0.19	1.09×10^{-1}	0.21	8.33×10^{-1}	0.20	4.34×10^{-2}
u8.51	8.51 – 8.51	0.54	1.08×10^{-7}	0.53	5.01×10^{-5}	0.53	1.16×10^{-4}
ADP	8.53 – 8.54	0.91	6.93×10^{-2}	0.80	8.64×10^{-3}	1.04	4.87×10^{-1}
ATP	8.54 – 8.55	0.56	2.90×10^{-9}	0.48	9.37×10^{-7}	0.67	1.20×10^{-3}
AMP	8.60 – 8.61	1.36	5.40×10^{-4}	1.39	3.18×10^{-2}	1.33	6.61×10^{-3}
Nicotinamide	8.71 – 8.73	0.80	1.03×10^{-1}	0.78	2.49×10^{-1}	0.80	8.44×10^{-2}

Table S3. Complete list of buckets with their FC values and *p*-values for comparison between male and female fish. For all buckets, the respective chemical shift range is shown.

		**Both Strains**		**HO5**		**iCab**	
**Bucket**	**Chemical Shift Range [ppm]**	**FC**	***p*-Value (FDR corr.)**	**FC**	***p*-Value (FDR corr.)**	**FC**	***p*-Value (FDR corr.)**
Leucine	0.96 – 0.98	0.91	1.67×10^{-1}	0.93	4.56×10^{-1}	0.89	2.84×10^{-1}
Isoleucine	1.01 – 1.03	0.97	6.65×10^{-1}	1.03	7.46×10^{-1}	0.92	4.73×10^{-1}
Valine	1.03 – 1.06	0.97	6.84×10^{-1}	1.04	6.14×10^{-1}	0.92	4.95×10^{-1}
Lactate/Threonine	1.32 – 1.35	1.00	9.86×10^{-1}	0.79	4.83×10^{-2}	1.15	2.48×10^{-1}
Alanine	1.47 – 1.50	0.80	2.18×10^{-3}	0.74	3.28×10^{-3}	0.86	1.13×10^{-1}
Proline	1.99 – 2.02	0.98	8.08×10^{-1}	1.00	9.66×10^{-1}	0.97	7.75×10^{-1}
Glu/Pro	2.02 – 2.07	0.97	5.39×10^{-1}	0.93	2.07×10^{-1}	1.00	9.46×10^{-1}
Glu/Gln/Met	2.10 – 2.14	0.87	1.11×10^{-3}	0.86	1.19×10^{-2}	0.88	4.25×10^{-2}
Glu/Pro/Malate	2.33 – 2.38	0.90	6.04×10^{-2}	0.84	1.25×10^{-2}	0.95	5.10×10^{-1}
u2.39	2.39 – 2.40	0.86	1.63×10^{-1}	1.07	6.14×10^{-1}	0.63	1.02×10^{-2}
Succinate	2.40 – 2.41	1.00	9.86×10^{-1}	0.89	5.50×10^{-2}	1.11	3.73×10^{-1}
Glutamine	2.44 – 2.48	0.80	5.15×10^{-5}	0.75	9.39×10^{-5}	0.85	5.79×10^{-2}
Glutathione	2.51 – 2.60	0.90	2.45×10^{-2}	0.87	1.21×10^{-2}	0.93	2.66×10^{-1}
Methionine	2.64 – 2.65	0.82	1.03×10^{-3}	0.84	2.76×10^{-2}	0.82	1.19×10^{-2}
Malate	2.65 – 2.66	0.96	6.84×10^{-1}	0.95	6.14×10^{-1}	0.98	8.91×10^{-1}
u2.71	2.71 – 2.72	1.12	1.22×10^{-1}	1.29	1.25×10^{-2}	0.95	5.69×10^{-1}
Sarcosine	2.74 – 2.74	0.88	1.73×10^{-1}	1.02	8.37×10^{-1}	0.79	9.54×10^{-2}
u2.76	2.75 – 2.76	0.88	6.61×10^{-2}	0.91	4.05×10^{-1}	0.84	5.79×10^{-2}
Aspartate	2.79 – 2.84	0.86	7.43×10^{-2}	0.92	4.05×10^{-1}	0.76	5.79×10^{-3}
Asparagine	2.85 – 2.89	0.96	4.46×10^{-1}	1.02	7.95×10^{-1}	0.89	1.51×10^{-1}
Lysine	3.01 – 3.03	0.84	1.11×10^{-3}	0.82	1.25×10^{-2}	0.85	5.79×10^{-2}
Creatine	3.03 – 3.04	0.42	3.98×10^{-12}	0.42	9.39×10^{-5}	0.42	1.64×10^{-7}
Ornithine	3.05 – 3.08	0.75	5.93×10^{-4}	0.75	1.45×10^{-2}	0.75	3.41×10^{-4}
Choline	3.20 – 3.21	0.88	1.40×10^{-1}	0.98	8.52×10^{-1}	0.82	1.07×10^{-1}
Phosphorylcholine	3.22 – 3.23	0.92	5.11×10^{-1}	0.77	1.94×10^{-1}	1.10	5.07×10^{-1}
Glycerophosphocholine	3.23 – 3.24	1.39	1.97×10^{-4}	1.19	1.40×10^{-1}	1.60	5.34×10^{-4}
Taurine/Betaine	3.27 – 3.28	1.13	1.02×10^{-3}	1.18	9.66×10^{-4}	1.08	1.46×10^{-1}
Hypotaurine	3.36 – 3.36	0.20	1.02×10^{-3}	0.20	4.33×10^{-2}	0.18	8.43×10^{-5}
Taurine	3.42 – 3.43	1.12	3.01×10^{-3}	1.16	9.07×10^{-3}	1.08	1.62×10^{-1}
Glycine	3.56 – 3.56	1.13	9.79×10^{-2}	1.10	4.63×10^{-1}	1.16	1.38×10^{-1}
Lactate	4.09 – 4.11	1.00	9.86×10^{-1}	0.95	3.35×10^{-1}	1.04	5.03×10^{-1}
u4.12/Lactate	4.11 – 4.12	1.13	2.07×10^{-2}	1.12	1.13×10^{-1}	1.14	1.13×10^{-1}
Glutathione_red	4.56 – 4.59	0.65	1.11×10^{-5}	0.76	1.14×10^{-2}	0.55	5.93×10^{-4}
Fumarate	6.52 – 6.52	0.89	4.21×10^{-1}	1.06	7.59×10^{-1}	0.58	9.54×10^{-2}
Histidine	7.11 – 7.12	0.95	4.21×10^{-1}	1.03	6.98×10^{-1}	0.88	1.57×10^{-1}
Tyrosine	7.18 – 7.22	0.66	1.91×10^{-4}	0.69	3.65×10^{-3}	0.61	5.26×10^{-3}
Phenylalanine	7.42 – 7.45	0.92	5.39×10^{-1}	1.05	7.64×10^{-1}	0.73	1.57×10^{-1}
3-Methylhistidine	8.05 – 8.06	12.77	2.63×10^{-12}	12.48	1.31×10^{-6}	13.00	1.46×10^{-5}
AXP	8.27 – 8.29	1.11	4.45×10^{-2}	1.02	7.93×10^{-1}	1.18	6.07×10^{-3}
Adenosine/Inosine	8.35 – 8.36	1.13	5.11×10^{-1}	1.34	1.53×10^{-1}	0.90	5.71×10^{-1}
Formate	8.46 – 8.46	0.16	1.03×10^{-3}	0.20	3.94×10^{-2}	0.16	6.07×10^{-3}
u8.51	8.51 – 8.51	1.46	2.80×10^{-3}	1.47	9.07×10^{-3}	1.47	6.07×10^{-3}
ADP	8.53 – 8.54	1.08	1.73×10^{-1}	0.94	5.31×10^{-1}	1.23	4.50×10^{-3}
ATP	8.54 – 8.55	1.23	7.49×10^{-2}	1.01	9.66×10^{-1}	1.39	5.26×10^{-3}
AMP	8.60 – 8.61	1.09	4.46×10^{-1}	1.10	5.52×10^{-1}	1.05	6.63×10^{-1}
Nicotinamide	8.71 – 8.73	1.57	1.03×10^{-3}	1.56	1.25×10^{-2}	1.59	3.43×10^{-2}
